# Clinical impact of CT-defined sarcopenia on long-term oncological outcomes in gastric cancer: validation of muscle mass cut-offs established according to EWGSOP recommendations

**DOI:** 10.1007/s10120-026-01796-x

**Published:** 2026-09-03

**Authors:** Jasmin Hasanovic, Paul Friedrich Esslinger, Veronika Lummer, Heiner Nebelung, Leo Misera, Ralf-Thorsten Hoffmann, Johanna Kirchberg, Marius Distler, Jürgen Weitz, Daniel E. Stange, Felix Merboth

**Affiliations:** 1 Department of Visceral, Thoracic, and Vascular Surgery, University Hospital Carl Gustav Carus, Technische Universität Dresden, Fetscherstrasse 74, 01307 Dresden, Germany; 2 National Center for Tumor Diseases (NCT/UCC), Dresden, Germany; 3 German Cancer Research Center (DKFZ), Heidelberg, Germany; 4 Faculty of Medicine Carl Gustav Carus, University Hospital, Technische Universität Dresden, Dresden, Germany; 5 Helmholtz-Zentrum Dresden-Rossendorf (HZDR), Dresden, Germany; 6 Faculty of Medicine, Institute and Polyclinic for Diagnostic and Interventional Radiology, University Hospital Carl Gustav Carus, Technische Universität Dresden, Dresden, Germany; 7 Institute of Radiology and Nuclear Medicine, Klinikum Nürnberg, Paracelsus Medical University, Nuremberg, Germany; 8 Else Kroener Fresenius Center for Digital Health, Technische Universität Dresden, Dresden, Germany

**Keywords:** Gastric cancer, Sarcopenia, Computed tomography, Skeletal muscle index, Psoas muscle thickness, Prognosis

## Abstract

**Background:**

Sarcopenia is an important prognostic factor in gastrointestinal malignancies, but CT-based definitions vary. This study evaluated recently published computed tomography (CT)-based cut-offs for skeletal muscle index (SMI) and psoas muscle thickness normalized to height (PMTH), derived from a young, presumably healthy reference population according to recommendations of the European Working Group on Sarcopenia in Older People (EWGSOP), in patients undergoing surgery for gastric cancer.

**Methods:**

This retrospective, single-center cohort study included patients who underwent surgery for gastric cancer between 2013 and 2018. Preoperative CT was used to assess SMI and PMTH. The recently published reference cut-offs were compared with the established definitions of Prado et al. (SMI) and Gu et al. (PMTH). Overall survival (OS) and recurrence-free survival (RFS) were the primary and secondary endpoints, respectively.

**Results:**

Data from 212 patients with complete 5-year follow-up were analyzed. The reference cut-offs significantly discriminated OS and RFS (SMI, both *p* < 0.001; PMTH, both *p* = 0.002), whereas the *established* cut-offs showed weaker or no discrimination. Patients with both low SMI and low PMTH had the worst OS and RFS (both *p* < 0.001). Combined low SMI and low PMTH was associated with worse OS in univariable analysis (HR 2.57, 95% CI 1.54–4.30, *p* < 0.001) and remained independently associated in multivariable analysis (HR 1.83, 95% CI 1.01–3.30; *p* = 0.046).

**Conclusions:**

The recently published CT-based reference cut-offs for SMI and PMTH provided consistent prognostic stratification after gastric cancer resection, and their combined use identified patients with particularly poor long-term survival.

**Electronic supplementary material:**

The online version of this article (https://doi.org/10.1007/s10120-026-01796-x) contains supplementary material, which is available to authorized users.

## Introduction

Gastric cancer remains among the major causes of cancer-related mortality worldwide. Approximately 1 million new cases are diagnosed annually, with the highest incidence observed in East Asia, Eastern Europe, and South America. Globally, gastric cancer is the fifth most common malignancy and the fourth leading cause of cancer-related mortality [1–3]. Although perioperative care and systemic therapies have advanced, overall survival (OS) remains limited and is closely associated with disease stage. For example, patients diagnosed with stage I disease have a five-year survival rate of approximately 75%, whereas those diagnosed with stage IV disease experience a decline in survival to < 10% [1, 4]. Surgical resection remains the cornerstone of curative treatment for localized tumors. However, the procedure is associated with considerable perioperative risk, with morbidity and mortality rates ranging from 20 to 40% and 2–5%, respectively [5, 6]. In cases of metastatic disease, systemic therapy is the primary treatment option, providing only modest survival improvements, despite recent therapeutic advances [4].

Given these challenges, improved risk stratification for gastric cancer is urgently needed, and relying solely on traditional staging systems is insufficient. Although traditional staging systems provide important prognostic information, they do not fully reflect tumor biology or patient-related factors that could influence outcomes [2]. One of these patient-related factors, sarcopenia, has emerged as a clinically relevant and independent determinant of outcomes in gastrointestinal malignancies, and is associated with worse OS and recurrence-free survival (RFS) across different gastrointestinal cancer types and treatment settings [7–9]. Beyond its negative impact on outcomes, sarcopenia is also highly relevant because of its considerable prevalence, with pooled estimates of approximately 33% in patients with gastrointestinal cancer and reported rates ranging from 14.7 to 43% depending on the study population and assessment method used [10–12]. This clinical relevance is also reflected in the current German guidelines for gastric cancer, which recommend optional computed tomography (CT)-based assessment of muscle mass at the third lumbar vertebra (L3) before curative treatment to estimate postoperative risk in patients diagnosed with gastric cancer [13].

In addition to its impact on survival, sarcopenia has been linked to higher rates of postoperative complications, including anastomotic leakage and surgical site infection(s), as well as prolonged hospital stays [14]. Furthermore, patients with sarcopenia often exhibit reduced tolerance to chemotherapy and immunotherapy, resulting in increased toxicity, treatment interruptions, and lower adherence rates [15].

Although the updated European Working Group on Sarcopenia in Older People (EWGSOP2) consensus defines sarcopenia as low muscle strength together with low muscle quantity or quality [16], most oncological studies assess reduced muscle mass and use the term “sarcopenia” accordingly. Despite the strong evidence supporting its clinical relevance, its definition remains inconsistent in the literature [17–19]. Most studies rely on CT-based measurements to calculate the Skeletal Muscle Index (SMI) at the level of L3, which correlates well with whole-body skeletal muscle mass and is the standard for body composition analysis in oncology [20–22]. In addition, parameters, such as the Psoas Muscle Index (PMI) and psoas muscle thickness per height (PMTH), have been proposed as complementary measures and have been associated with survival outcomes in patients with gastrointestinal cancer [21, 23]. However, the proposed cut-off values for SMI and PMTH differed substantially among populations and study designs. This variability makes it difficult to compare different studies and, consequently, raises concerns regarding generalizability. As a result, the reported prevalence and prognostic impact of sarcopenia vary considerably among cohorts [24].

Therefore, the aim of the present study was to validate the CT-based cut-offs for the SMI and PMTH, derived from a young, presumably healthy Caucasian reference population in accordance with the EWGSOP recommendations [25], hereafter referred to as the reference cut-offs. As these thresholds were established independently of oncological outcomes, validation in clinical populations is required. Because muscle strength and physical performance were not assessed, the present study evaluated only the CT-derived muscle-mass component and not the complete EWGSOP2 definition of sarcopenia. We therefore compared the prognostic performance of recently published reference cut-offs with established cut-off definitions proposed by Prado et al. for SMI [22] and by Gu et al. for PMTH [23] in patients undergoing curative-intent resection for gastric cancer.

## Methods

### Study design and patient population

This retrospective, single-center cohort study included data from consecutive patients who underwent curative-intent surgical resection for histologically confirmed gastric cancer at the Department of Visceral, Thoracic, and Vascular Surgery, University Hospital Carl Gustav Carus (Dresden, Germany), between 2013 and 2018. All patients were treated according to current standards and were followed up for 5 years. The study protocol was approved by the institutional review board (BO-EK-109032022). All procedures conformed to the institutional standards and principles of the Declaration of Helsinki. The reference cut-offs applied here were derived from an independent cohort of young, presumably healthy Caucasian individuals [25]. The derivation cohort originated from the same institution as the present study. However, no patients overlapped between the two cohorts. Thus, the validation was independent at the participant level, although both cohorts originated from the same institution.

Eligible patients were ≥ 18 years of age, had histologically confirmed gastric adenocarcinoma and underwent curative-intent surgical resection at the authors’ institution between 2013 and 2018. Complete perioperative clinical documentation, preoperative imaging suitable for the assessment of skeletal muscle mass, and follow-up data were prerequisites for inclusion in the final study cohort. Patients with a second primary malignancy, those in whom surgery was performed under emergency conditions, or those missing essential clinical or imaging data required for analysis were excluded. Patients lost to follow-up were also excluded from the final analysis.

### Surgical procedures

Surgical procedures included subtotal, total, and extended transhiatal gastrectomy. The operations were performed using either an open or a minimally invasive approach (laparoscopic or robotic), depending on patient characteristics and surgeon preference.

### Data collection

Demographic, clinical, perioperative, and pathological data were extracted from institutional electronic medical records. Tumor characteristics, including pathological T category (pT), pathological nodal category (pN), metastatic status (M), resection status, and Union for International Cancer Control (UICC) stage, were obtained from pathology reports. Information regarding perioperative therapy, surgical procedures, postoperative complications, and mortality was prospectively documented in the institutional database and retrospectively analyzed.

### Assessment of skeletal muscle mass and definition of sarcopenia

Preoperative CT scans performed during routine staging were used to assess skeletal muscle mass. When multiple CT scans were available, data from the scan closest to the date of surgery were analyzed.

The skeletal muscle area (SMA [cm^2^]) was measured on axial CT images at the level of the third lumbar vertebra (L3). Segmentation of the skeletal muscle was performed automatically using a pretrained nnU-Net (version 2.2.1) segmentation model [26]. The segmentation masks were visually inspected by a board-certified radiologist and cases with inaccurate segmentation masks were excluded. The SMI (cm^2^/m^2^) was calculated by normalizing the SMA to patient height squared.

Psoas muscle thickness was measured on axial CT images at the level of the umbilicus. The transverse diameter of the psoas muscle was measured perpendicular to its longest axis. Psoas muscle thickness was normalized to patient height to calculate the psoas muscle thickness normalized to height (PMTH [mm/m]) [23, 27, 28].

Sarcopenia was classified using previously published cut-off values for SMI and PMTH [22, 23], as well as the recently published CT-based reference cut-offs established according to the EWGSOP recommendations [25]. According to these reference thresholds, sarcopenia was defined as SMI < 33.2 cm^2^/m^2^ for women and < 44.0 cm^2^/m^2^ for men, and PMTH < 15.7 mm/m for women and < 20.2 mm/m for men [25]. These thresholds were compared with previously published cut-off values proposed by Prado et al. [22] for SMI (SMI < 38.5 cm^2^/m^2^ for women and < 52.4 cm^2^/m^2^ for men) and by Gu et al. [23] for PMTH (sex-nonspecific PMTH < 16.8 mm/m).

### Study endpoints

The primary endpoint of the study was OS, defined as the time from surgery to death from any cause. The main objective of this study was to evaluate the prognostic performance of the CT-based reference cut-offs by comparing them with previously established thresholds (SMI according to Prado et al. [22] and PMTH according to Gu et al. [23]). In addition, we assessed whether the combined evaluation of SMI and PMTH improved prognostic stratification and whether combined sarcopenia remained independently associated with OS in multivariable analysis. Secondary endpoints included RFS, defined as the time from surgery to the first documented tumor recurrence or death, whichever occurred first. Postoperative outcomes, including morbidity, mortality, and major complications, were also evaluated.

### Statistical analysis

Statistical analyses were performed using SPSS version 28.0 (IBM Corp., Armonk, NY, USA) and Python (version 3.12.1; Python Software Foundation, Beaverton, NY, USA). Continuous variables are expressed as mean ± standard deviation or median with interquartile range (IQR), while categorical variables are reported as count and percentage. Group comparisons were performed using the chi-squared or Fisher’s exact test for categorical variables and the Student’s *t*-test or Mann–Whitney U test for continuous variables, as appropriate.

OS and RFS rates were analyzed using the Kaplan–Meier method and compared using the log-rank test. Univariate and multivariate Cox proportional hazards regression analyses were used to identify independent prognostic factors. As an exploratory sensitivity analysis, OS was examined among neoadjuvant-treated patients according to subsequent receipt of adjuvant chemotherapy. Subgroup-specific Cox models were performed, and effect modification was assessed using a sarcopenia × adjuvant chemotherapy interaction term. Recurrence endpoints were analyzed using both Fine–Gray sub-distribution hazard models to account for competing risks and cause-specific Cox proportional hazards models. All statistical tests were two-sided and differences with *p* < 0.05 were considered to be significant. No formal adjustment for multiple testing was applied despite evaluation of multiple cut-off definitions for OS, RFS, and postoperative outcomes. Secondary findings should therefore be interpreted cautiously, and recurrence-pattern analyses should be regarded as exploratory and hypothesis-generating.

## Results

### Study cohort

Data from 243 patients who underwent surgery for gastric cancer during the study period were analyzed. After excluding 31 patients due to a second malignancy, emergency surgery, missing data, or loss to follow-up, data from 212 constituted the final cohort for postoperative outcome analyses. For survival analyses, imaging availability permitted the inclusion of 200 patients for SMI-based analyses and 203 for PMTH-based analyses.

The median age of the study population was 69.8 years (IQR 61.7–77.9 years), and 31.6% of patients were female. The median body mass index (BMI) was 25.3 kg/m^2^ (IQR 22.9–27.8 kg/m^2^). More than one-half of patients received neoadjuvant chemotherapy (53.8%), with fluorouracil, leucovorin, oxaliplatin, docetaxel (FLOT) (54.4%) and epirubicin, cisplatin, and 5-fluorouracil (ECF) (24.6%) the most commonly used regimens. In contrast, only 31.1% of patients received adjuvant chemotherapy, most frequently FLOT (54.5%) or ECF (19.7%). Regarding tumor characteristics, most patients presented with advanced disease, with UICC stage III or IV in 50% of cases. Pathological nodal metastases were present in 57.1% of patients and R0 resection was achieved in 88.2% (Table 1).

**Table 1:** Baseline and demographic characteristics (*n* = 212)

Variable	*n* = 212
*Gender*	
Female (n, %)	67 (31.6)
Male (n, %)	145 (68.4)
Age (years, median, IQR)	69.75 (61.70–77.90)
BMI (kg/m^2^, median, IQR)	25.32 (22.87–27.77)
*Risk factors*	
Nicotine abuse (n, %)	49 (23.1)
Alcohol abuse (n, %)	11 (5.2)
Diabetes mellitus (n, %)	54 (25.5)
*ASA classification*	
ASA I (n, %)	7 (3.3)
ASA II (n, %)	78 (36.8)
ASA III (n, %)	122 (57.5)
ASA IV (n, %)	5 (2.4)

Abbreviations: *BMI* Body mass index, *ASA* American Society of Anesthesiologists, *IQR* interquartile range

### Surgical procedures and postoperative outcomes

Total gastrectomy was performed in 48.1% of patients, subtotal gastrectomy in 24.5%, and extended transhiatal gastrectomy in 27.4%. Overall, postoperative complications occurred in 54.7% of patients, whereas major complications (Clavien–Dindo > IIIa) occurred in 21.7%. Anastomotic leakage was observed in 9.9% of cases, pneumonia in 8.5%, and respiratory insufficiency in 16.5%. In-hospital and 30-day mortality were 7.5% and 5.2%, respectively (Table 2).

**Table 2:** Tumor characteristics, treatment, surgical procedures, and postoperative outcomes (*n* = 212)

Variable	n = 212
*Perioperative chemotherapy*	
Neoadjuvant chemotherapy (n, %)	114 (53.8)
FLOT (n, %)	62 (54.4)
ECF (n, %)	28 (24.6)
Other (n, %)	24 (21)
Adjuvant chemotherapy (n, %)	66 (31.1)
FLOT (n, %)	36 (54.5)
ECF (n, %)	13 (19.7)
Other (n, %)	17 (25.7)
*UICC stage*	
UICC stage I (n, %)	65 (30.7)
UICC stage II (n, %)	41 (19.3)
UICC stage III (n, %)	64 (30.2)
UICC stage IV (n, %)	42 (19.8)
*pT category*	
pT0 (n, %)	11 (5.2)
pT1 (n, %)	45 (21.2)
pT2 (n, %)	25 (11.8)
pT3 (n, %)	95 (44.8)
pT4 (n, %)	36 (17.0)
*pN category*	
pN0 (n, %)	91 (42.9)
pN1 (n, %)	44 (20.8)
pN2 (n, %)	33 (15.6)
pN3 (n, %)	44 (20.8)
*Metastatic status*	
M0 (n, %)	174 (82.1)
M1 (n, %)	38 (17.9)
*Resection status*	
R0 (n, %)	187 (88.2)
R1/R2 resection (n, %)	25 (11.8)
*Surgical procedure*	
Subtotal gastrectomy (n, %)	52 (24.5)
Total gastrectomy (n, %)	102 (48.1)
Extended transhiatal gastrectomy (n, %)	58 (27.4)
*Postoperative complications*	
Overall postoperative complications (n, %)	116 (54.7)
Major complications, Clavien-Dindo grade > IIIa (n, %)	46 (21.7)
*Type of postoperative complication* (n, %)	
Anastomotic leakage (n, %)	21 (9.9)
Duodenal stump leakage (n, %)	11 (5.2)
Organ/space infection (n, %)	26 (12.3)
Surgical site infection (n, %)	27 (12.7)
Pneumonia (n, %)	18 (8.5)
Respiratory insufficiency (n, %)	35 (16.5)
Other pulmonary complications (n, %)	45 (21.2)
*Mortality*	
In-hospital mortality (n, %)	16 (7.5)
30-day mortality (n, %)	11 (5.2)

Abbreviations: *BMI* Body mass index, *ASA* American society of anesthesiologists, *IQR* Interquartile range, *UICC* Union for international cancer control, *CD* Clavien–dindo classification

### Prevalence of sarcopenia

The proportion of patients classified as sarcopenic varied substantially, depending on the definition applied. Using previously published cut-off values, sarcopenia was identified in 119 (59.5%) patients based on SMI (Prado et al. [22]) and 48 (23.7%) based on PMTH (Gu et al. [23]). In contrast, the reference cut-off values classified 36 (18.0%) patients as sarcopenic based on SMI and 81 (39.9%) based on PMTH (Supplementary Table 1).

### Cut-off discrimination: OS

**Fig. 1:**
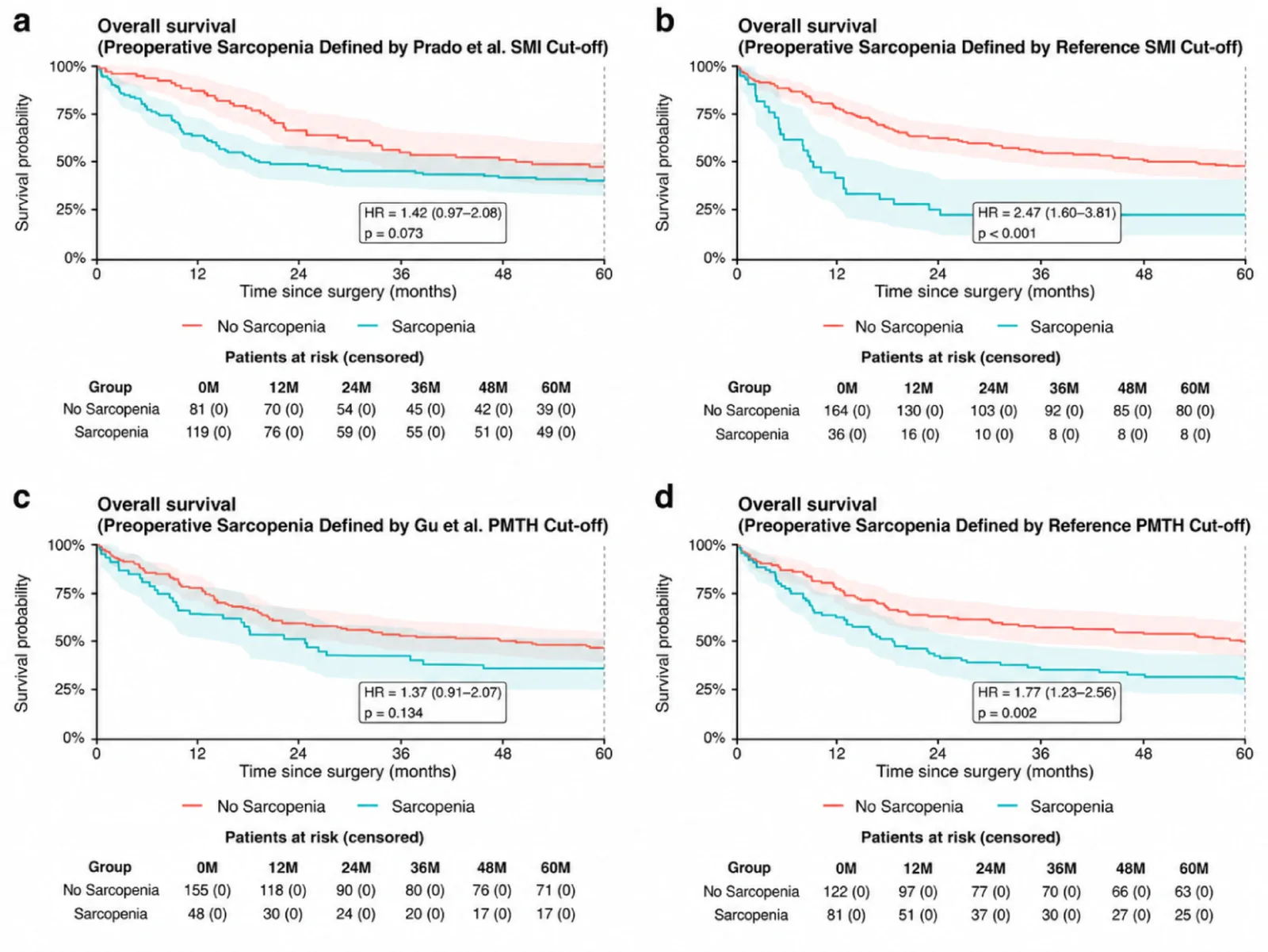
Kaplan–Meier analysis of overall survival according to preoperative sarcopenia by different definition

OS differed significantly depending on cut-off value. SMI-defined sarcopenia based on the Prado et al. cut-offs showed a non-significant trend toward worse OS (log-rank *p* = 0.073) (Fig. 1a). In contrast, the reference SMI cut-offs clearly separated the survival curves and identified a significantly worse OS in patients with sarcopenia (log-rank *p* < 0.001) (Fig. 1b). For PMTH-defined sarcopenia, the Gu et al. cut-off did not significantly distinguish OS (log-rank *p* = 0.129) (Fig. 1c), whereas the *reference* PMTH cut-offs showed a significant difference (log-rank *p* = 0.002) (Fig. 1d).

### Cut-off discrimination: RFS

**Fig. 2:**
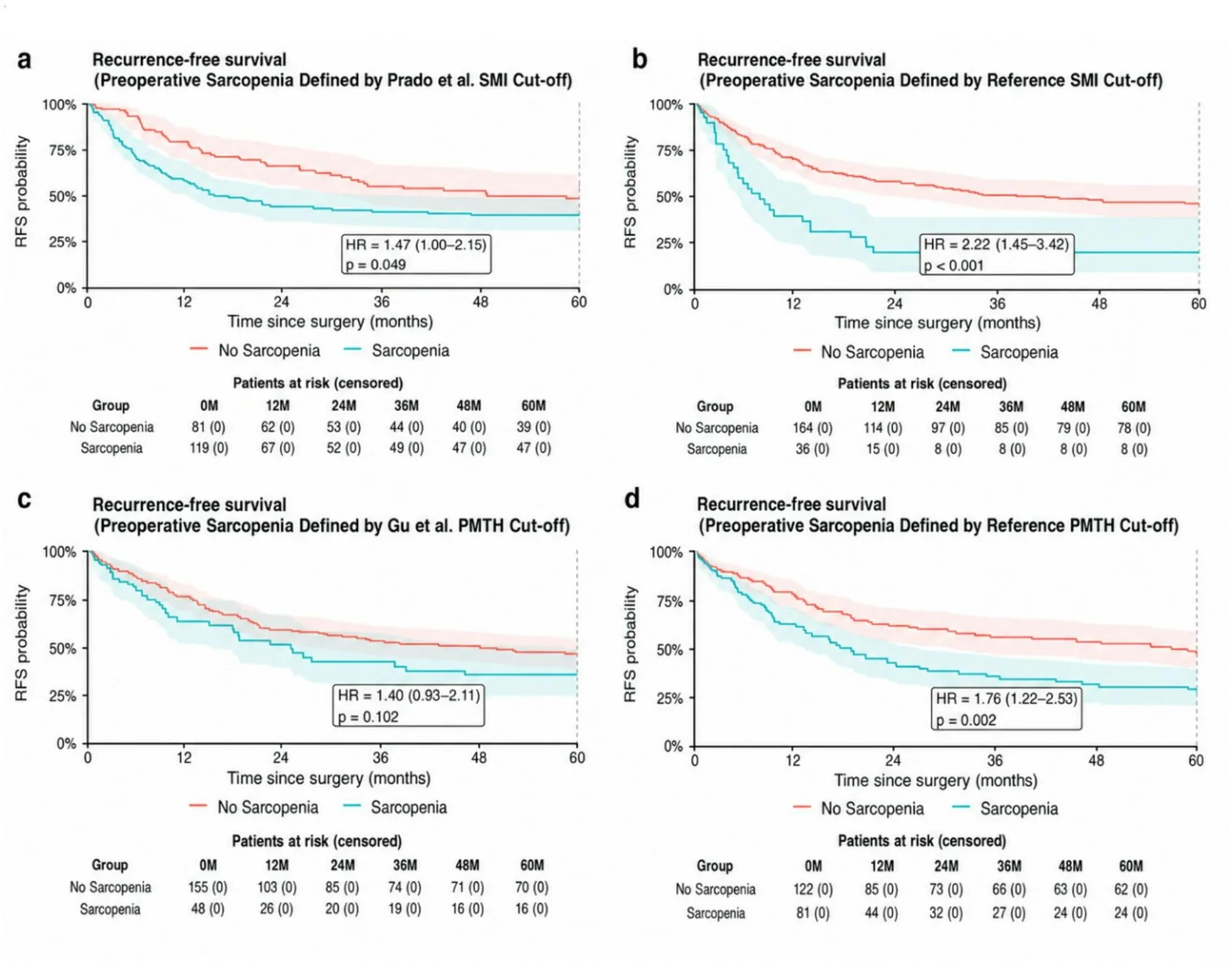
Kaplan–Meier analysis of recurrence-free survival according to preoperative sarcopenia by different definition

A similar, albeit less uniform, pattern was observed for RFS. The SMI cut-off according to Prado et al. significantly separated RFS (log-rank *p* = 0.049) (Fig. 2a); however, the reference SMI cut-off more clearly separated RFS curves (log-rank *p* < 0.001) (Fig. 2b). According to Gu et al. [23], the PMTH cut-off did not significantly separate RFS (log-rank *p* = 0.113) (Fig. 2c), whereas the reference PMTH cut-off showed a significant separation of the RFS curves (log-rank *p* = 0.002) (Fig. 2d).

### Added utility of combined SMI and PMTH definitions

**Fig. 3:**
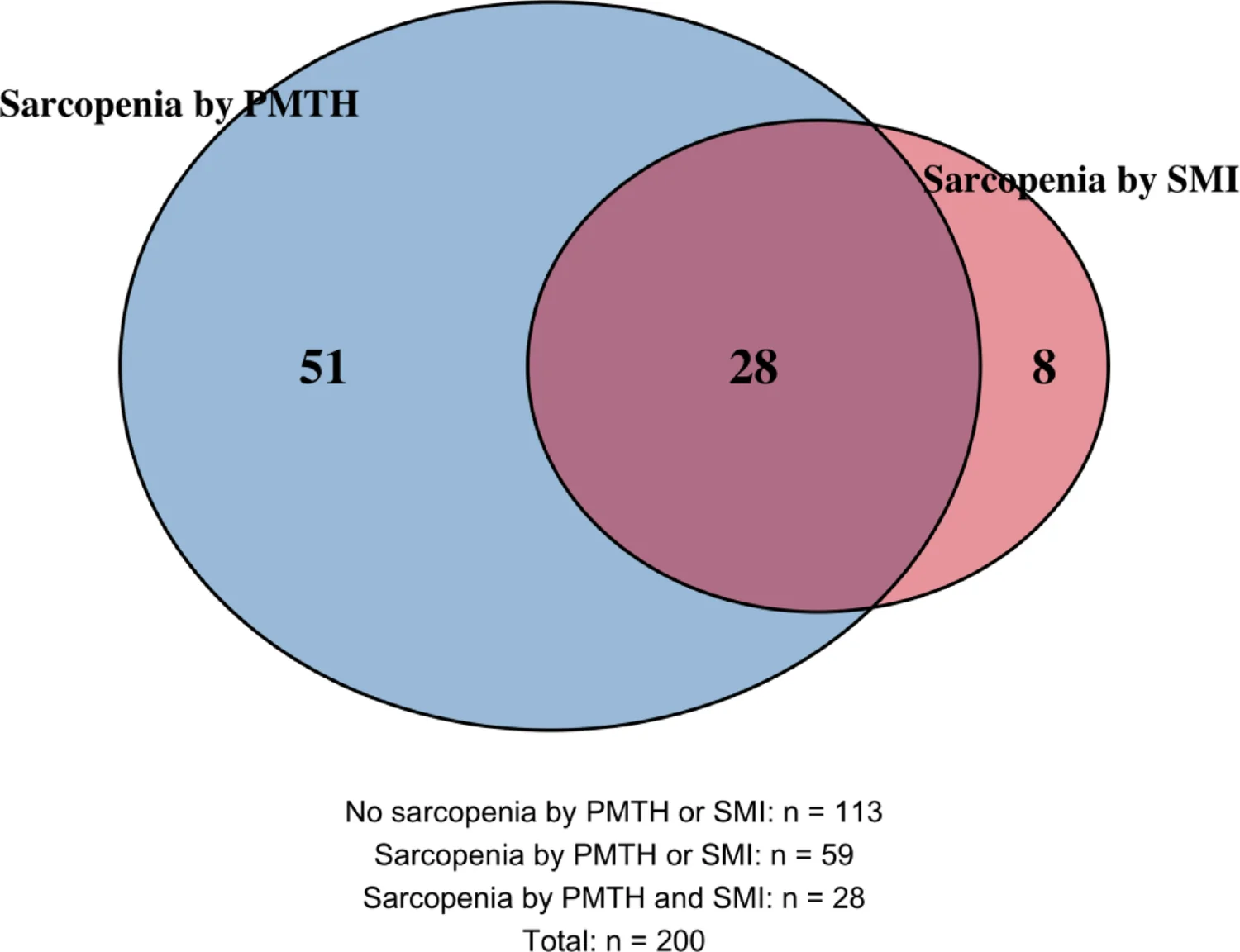
Overlap between reference PMTH- and SMI-defined sarcopenia. Venn diagram showing the overlap between sarcopenia defined by psoas muscle thickness normalized to height (PMTH) and skeletal muscle index (SMI) according to the *reference* cutoff values in 200 patients. Fifty-one patients were classified as sarcopenic by PMTH only, 8 by SMI only, and 28 by both parameters, while 113 patients were classified as non-sarcopenic by both definitions

**Fig. 4:**
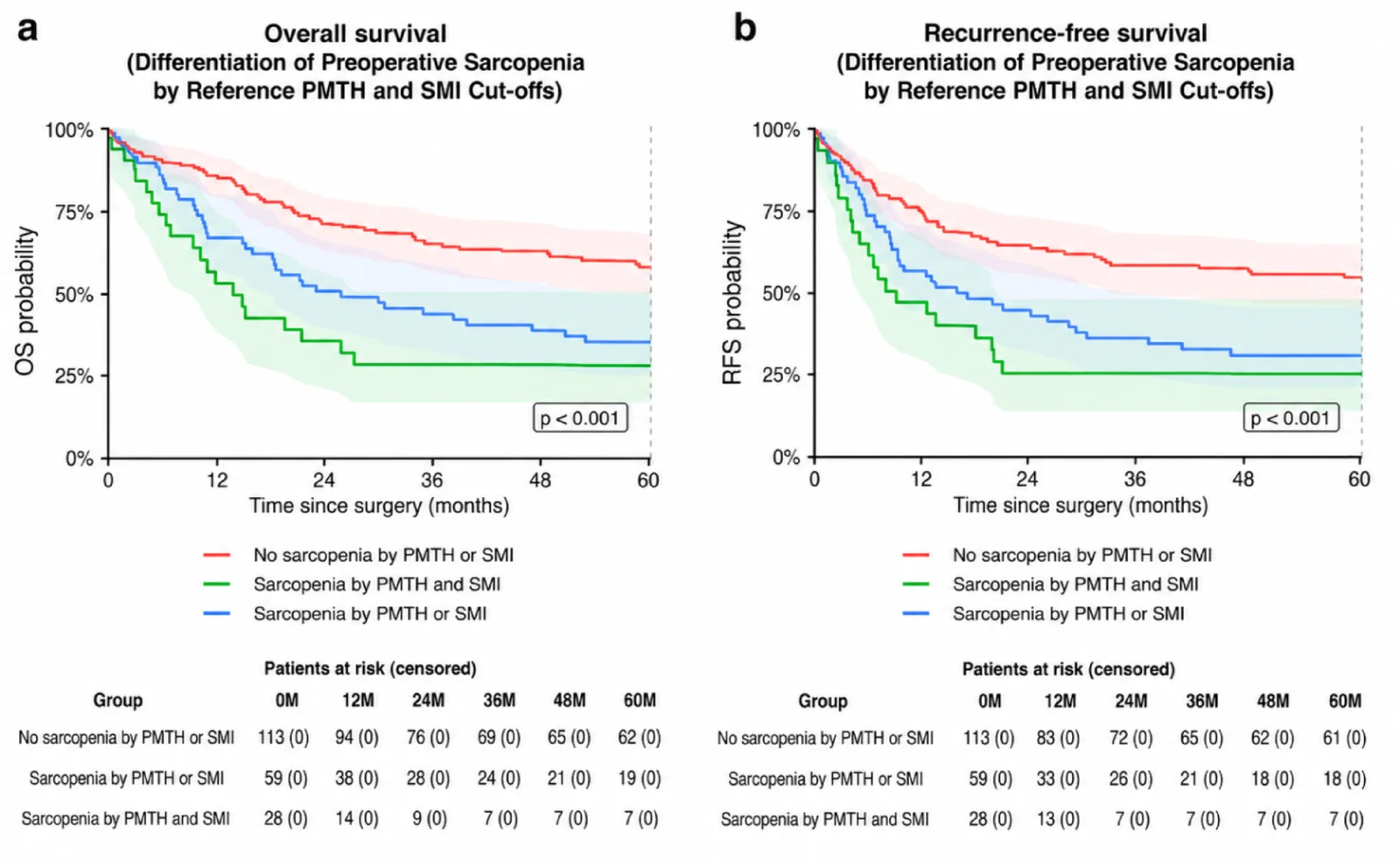
Kaplan–Meier analyses of overall survival and recurrence-free survival according to preoperative sarcopenia status groups based on the reference SMI and PMTH cut-offs

Of 200 patients, 28 were classified as sarcopenic by both SMI and PMTH, 51 by PMTH alone, and 8 by SMI alone, whereas 113 were classified as non-sarcopenic by both definitions. This overlap is illustrated in a Venn diagram based on the reference cut-offs (Fig. 3). The combination of SMI and PMTH resulted in a clearer separation of the prognostic groups. In the three-level classification (both positive, only one positive, and both negative), OS differed significantly between the groups (log-rank *p* < 0.001) (Fig. 4a), showing a stepwise decrease in survival from patients without sarcopenia to those with sarcopenia in one parameter and from those with sarcopenia in both parameters. A similar pattern was observed for RFS (log-rank test *p* < 0.001) (Fig. 4b).

Using a pragmatic binary combined definition (≥ 1 parameter positive vs. both negative), OS also differed significantly between the groups (log-rank *p* < 0.001) (Supplementary Fig. 1a), and the same was observed for RFS (log-rank *p* < 0.001) (Supplementary Fig. 1b).

### Overall survival

To further evaluate whether sarcopenia defined by the reference cut-offs provided prognostic information beyond established clinicopathological factors, Cox proportional hazards regression analyses for OS were performed. In univariate analysis, higher UICC stage (HR 1.95, 95% CI 1.63–2.32; *p* < 0.001), lower BMI (HR 0.93 per kg/m^2^, 95% CI 0.88–0.97; *p* = 0.002), and combined sarcopenia positivity for both cut-offs (HR 2.57, 95% CI 1.54–4.30; *p* < 0.001) were associated with worse OS. Age showed a borderline association (HR 1.02 per year, 95% CI 1.00–1.03; *p* = 0.059), whereas sex, ASA status, and major postoperative complications were not statistically significant.

In multivariable analysis including age, BMI, UICC stage, and combined sarcopenia, lower BMI (HR 0.90, 95% CI 0.84–0.96; *p* = 0.002) and higher UICC stage (HR 1.93, 95% CI 1.54–2.41; *p* < 0.001) remained independently associated with worse OS. Combined sarcopenia positivity for reference cut-offs also remained an independent prognostic factor (HR 1.83, 95% CI 1.01–3.30; *p* = 0.046) (Table 3). In the exploratory sensitivity analysis among neoadjuvant-treated patients, sarcopenia was associated with increased mortality risk in both treatment groups across all definitions, with no evidence that this association differed according to subsequent adjuvant chemotherapy (all p for interaction ≥ 0.590) (Supplementary Fig. 2). Kaplan-Meier analyses and subgroup-specific Cox models for overall survival with respect to perioperative therapy showed no difference compared with the unadjusted analyses (Supplementary Figs. 3, 4 and 5).

**Table 3:** Univariate and multivariable Cox regression analyses for overall survival using the combined sarcopenia definition based on reference SMI and PMTH cut-offs

Variable	Univariate HR (95% CI)	*p*-value	Multivariate HR (95% CI)	*p*-value
Age (per year)	1.02 (1.00–1.03)	0.059	1.01 (0.98–1.03)	0.531
Sex (male vs. female)	1.06 (0.72–1.56)	0.773	–	–
ASA status (III–IV vs. I–II)	1.27 (0.88–1.84)	0.206	–	–
Major postoperative complications (Clavien–Dindo > IIIa vs. no)	1.37 (0.95–1.97)	0.089	–	–
UICC stage (per one-stage increase)	1.95 (1.63–2.32)	**< 0.001**	1.93 (1.54–2.41)	**< 0.001**
BMI (per kg/m^2^)	0.93 (0.88–0.97)	**0.002**	0.90 (0.84–0.96)	**0.002**
Sarcopenia (PMTH and SMI) vs. no sarcopenia	2.57 (1.54–4.30)	**< 0.001**	1.83 (1.01–3.30)	**0.046**

Bold values are significant

Abbreviations: *HR* Hazard ratio, *CI* Confidence interval, *BMI* Body mass index, *UICC* Union for international cancer control, *PMTH* Psoas muscle thickness normalized to height, *SMI* Skeletal muscle index, bold = significant

### Recurrence patterns and competing-risk analysis

To further characterize recurrence patterns, competing risk analyses were performed for the combined sarcopenia definition based on both reference SMI and PMTH cut-offs. In the cause-specific Cox regression analysis, sarcopenia was associated with a significantly higher risk for overall recurrence (hazard ratio [HR] 2.34 [95% CI 1.128–4.854]; *p* = 0.0225) (Table 4).

**Table 4:** Cause-specific Cox and Fine–Gray regression analyses for overall recurrence and recurrence patterns using the combined sarcopenia definition based on reference SMI and PMTH cut-offs

Endpoint	CSC events (*n*)	HR	95% CI	*p*-value	Fine–Gray events (*n*)	sHR	95% CI	*p*-value
Overall recurrence	68	2.340	1.128–4.854	0.023	37	1.737	0.825–3.658	0.146
*Recurrence pattern*								
Hematogenous recurrence	37	3.491	1.010–12.066	0.048	11	2.509	0.732–8.593	0.143
Lymphatic recurrence	25	0.693	0.087–5.501	0.729	14	0.672	0.149–3.033	0.605
Local recurrence	18	0.796	0.099–6.378	0.829	9	0.501	0.062–4.039	0.517
Peritoneal recurrence	34	9.116	2.012–41.303	0.004	15	1.526	0.487–4.784	0.468
Other recurrence	5	NA	NA	NA	3	NA	NA	NA

Abbreviations: *CI* Confidence interval, *HR* Hazard ratio, *sHR* Subdistribution hazard ratio

Cause-specific Cox regression and Fine–Gray competing-risk regression were performed for overall recurrence and recurrence subtypes according to the combined reference-population sarcopenia definition. Death was treated as a competing event in Fine–Gray analyses. Event counts are shown separately for each modeling approach

In contrast, the Fine–Gray model did not reveal a statistically significant association with the sub-distribution hazard of recurrence (subdistribution HR 1.737 [95% CI 0.825–3.658]; *p* = 0.146). Exploratory subtype analyses in the cause-specific model suggested an association particularly with peritoneal (HR 9.116 [95% CI 2.012–41.303]; *p* = 0.004) and hematogenous (HR 3.491 [95% CI 1.01–12.066]; *p* = 0.048) recurrence, whereas associations for other recurrence patterns were less consistent. Fine–Gray analyses of the recurrence subtypes were not statistically significant (Table 4). These subtype analyses were based on few events and are therefore imprecise, as reflected by the wide confidence intervals. They should be interpreted with caution and regarded as exploratory.

### Baseline characteristics associated with sarcopenia

Multivariable logistic regression (*n* = 200) showed that sarcopenia was independently associated with older age (adjusted OR 2.808, 95% CI 1.468–5.374; *p* = 0.002) and advanced tumor stage (adjusted OR 3.883, 95% CI 2.030–7.427; *p* < 0.001), whereas higher BMI was inversely associated with sarcopenia (adjusted OR 0.389, 95% CI 0.210–0.720; *p* = 0.003) (Supplementary Table 2).

Consistently, sarcopenic patients were older, had a lower BMI, and more often had advanced tumor stage (Supplementary Table 3).

In the subgroup positive for both reference cut-offs, age and BMI remained significantly different, whereas tumor stage variables were not significant (Supplementary Table 4).

### Postoperative outcomes

Sarcopenia was not consistently associated with overall postoperative or major complications across definitions. Significant associations were limited to respiratory insufficiency for SMI-defined sarcopenia according to Prado et al. (*p* = 0.013) and pneumonia for PMTH-defined sarcopenia according to Gu et al. (*p* = 0.041) (Supplementary Table 5).

The CCI did not differ significantly according to any sarcopenia definition (Supplementary Table 6). Length of hospital stay differed significantly for PMTH-based sarcopenia (Gu et al., *p* = 0.035; reference cut-offs, *p* = 0.018) (Supplementary Table 7).

## Discussion

The present study evaluated whether the recently published reference cut-offs for CT-defined sarcopenia are suitable for prognostic stratification in patients with gastric carcinoma undergoing curative-intent surgery, and whether they provide better prognostic discrimination than the commonly used CT-based sarcopenia cut-offs.

Over the past decade, sarcopenia has become an important prognostic marker for patients undergoing surgery for gastric cancer. Multiple cohort studies and meta-analyses have demonstrated that preoperative sarcopenia is associated with impaired OS and disease-free survival after gastrectomy [29–32]. Despite its well-established clinical relevance, its definition remains unclear [17–19]. The same issue was also evident in the present study given that the prevalence of sarcopenia varied considerably depending on the cut-off values used. This variation likely reflects the different methodological approaches used to define cut-off values. The commonly used SMI cut-offs proposed by Prado et al. were derived by outcome-based optimal stratification, whereas the CT-based reference cut-offs were established in a young and healthy Caucasian cohort and defined as 2 standard deviations below the sex-specific mean, in accordance with the EWGSOP recommendations. Similarly, the PMTH cut-off proposed by Gu et al. was derived by receiver operating characteristic curve analysis to predict SMI-defined sarcopenia rather than by direct survival-based stratification. These methodological differences may, at least in part, explain why the prevalence of sarcopenia differed markedly depending on the cut-off value applied. Accordingly, when applying the SMI cut-off values according to Prado et al., a large proportion of patients were classified as sarcopenic, whereas a much smaller subgroup of patients was classified as sarcopenic when the reference SMI cut-offs were used. For PMTH, the opposite pattern was observed, with more patients classified as sarcopenic according to the reference cut-offs than according to the Gu et al. cut-off. These findings suggest that both measures may reflect related, but not identical aspects of body composition and, are therefore, consistent with previous studies reporting that psoas-based measures are not fully interchangeable with SMIs [33].

From a clinical perspective, one of the most relevant findings of the present study was that the association between sarcopenia and oncological outcomes varied depending on the cut-off values applied. In our cohort, the reference cut-off values consistently identified subgroups with less favorable OS and RFS than the established Gu et al. cut-off, whereas the Prado et al. cut-off also demonstrated prognostic relevance for RFS. However, because the Prado et al. cut-off classifies a substantially larger proportion of patients as sarcopenic than the reference *SMI* cut-off, it may be less selective in identifying a distinctly high-risk subgroup in this gastric cancer population.

The discrimination between OS and RFS was most obvious when reference SMI and PMTH cut-offs were combined because patients classified as sarcopenic by both parameters had the poorest OS and RFS. This clinical relevance of combined sarcopenia definition was further strengthened by Cox regression analyses. In univariate analysis, both SMI- and PMTH-based sarcopenia according to reference cut-offs, as well as the combined definition, were associated with worse OS. Combined sarcopenia remained independently associated with OS after adjustment for age, BMI, and UICC stage. This was supported by the sensitivity analysis, which showed an increased mortality risk associated with sarcopenia in both treatment groups, irrespective of subsequent adjuvant chemotherapy. Given the previous evidence suggesting that more pronounced muscle depletion is associated with worse oncological outcomes [34], it can be assumed that patients classified as sarcopenic by both parameters had more severe muscle loss and lower physical reserve. Consequently, the combination of SMI and PMTH may complement risk stratification.

To further analyze recurrence, competing-risk analyses were performed using the combined sarcopenia definition based on the reference cut-offs. In these models, combined sarcopenia was associated with a significantly higher cause-specific hazard of recurrence, whereas the Fine–Gray model did not demonstrate a statistically significant increase in subdistribution hazard. This suggests that sarcopenia may influence recurrence dynamics among patients who remain alive and recurrence-free, whereas its effect on cumulative recurrence incidence is attenuated when competing events, particularly death before recurrence, are considered. Exploratory subtype analyses suggested associations with peritoneal and hematogenous recurrence. However, the small event numbers and wide confidence intervals limit the reliability of these estimates.

Consistent with a previously published meta-analysis [10], results of the present study revealed that sarcopenia was independently associated with older age, lower BMI, and advanced tumor stage in multivariable logistic regression analysis. In contrast to the clear findings regarding OS and RFS, the association between sarcopenia and postoperative morbidity was less consistent. Despite robust evidence in the literature supporting this relationship [14, 35], sarcopenia was not consistently associated with overall postoperative complications in this study. One possible explanation is that the different cut-off definitions produced markedly different group sizes, which may have limited the ability to detect consistent associations with postoperative outcomes.

The present study has several limitations, including its retrospective single-center design, the potential for selection bias, and the lack of data on muscle strength and functional parameters. Therefore, sarcopenia was defined solely based on imaging-derived muscle mass. Accordingly, the present findings validate only CT-derived muscle-mass cut-offs derived according to the EWGSOP reference-population methodology and should not be interpreted as validation of the complete EWGSOP2 diagnostic algorithm. Nevertheless, CT-based muscle assessment remains the current standard for oncological body composition analysis [36] Performance status (ECOG/WHO) was not systematically recorded in this retrospective cohort and could therefore not be included in the analysis. Consequently, residual confounding by functional status cannot be excluded. Although ASA physical status was available, it cannot fully substitute for an oncological performance-status measure. Because adjuvant therapy was assessed as a post-baseline binary exposure and detailed information on treatment timing and completion was unavailable, the exploratory treatment-stratified analysis may be subject to guarantee-time bias. Finally, no formal adjustment for multiple testing was applied. Secondary and exploratory findings should therefore be interpreted cautiously.

Despite these limitations, our findings support the clinical relevance of the reference cut-offs in gastric cancer. If validated in external cohorts, these thresholds may contribute to more standardized risk stratification and help identify patients who could benefit from nutritional and perioperative supportive interventions, including prehabilitation [37–39].

## Conclusion

The CT-based sarcopenia cut-offs derived from a healthy reference population according to the EWGSOP recommendations provided consistent prognostic stratification in patients undergoing surgery for gastric cancer. Although the SMI cut-off proposed by Prado et al. also showed prognostic relevance for RFS, the reference cut-offs showed the most consistent pattern across survival endpoints in this cohort. The combined assessment of SMI and PMTH identified patients with the poorest long-term outcomes and remained independently associated with worse overall survival in multivariable analysis. These findings support further external validation of the reference cut-offs.

## Electronic supplementary material

Below is the link to the electronic supplementary material.

Supplementary file 1
