# Supplementary material for: Clinical impact of CT-defined sarcopenia on long-term oncological outcomes in gastric cancer: validation of muscle mass cut-offs established according to EWGSOP recommendations
Source: Gastric Cancer. 2026 Sep 3;29(6):1337–48. doi: 10.1007/s10120-026-01796-x (PMC13754937; doi:10.1007/s10120-026-01796-x)
Supplement: Supplementary file 1 [file 10120_2026_1796_MOESM1_ESM.docx]

**Supplementary tables and figures**

**Supplementary Table 1. Prevalence of sarcopenia**

| **Variable** | **n (%)** |
| --- | --- |
| SMI (Prado et al.) | 119 (59.5) |
| SMI (Reference cut-off) | 36 (18.0) |
| PMTH (Gu et al.) | 48 (23.7) |
| PMTH (Reference cut-off) | 81 (39.9) |

**Abbreviations**: SMI, skeletal muscle index; PMTH, psoas muscle thickness,

**Supplementary table 2. Univariate and multivariable logistic regression analysis of factors associated with sarcopenia in the study cohort (n=200)**

| Preoperative  variable | Sarcopenia negative (n=117) | Sarcopenia positive (n=88) | Univariate p | Univariate OR (95% CI) | Multivariate p | Adjusted OR (95% CI) |
| --- | --- | --- | --- | --- | --- | --- |
| Gender |  |  | 0.169 | 1.585 (0.860–2.921) | — | — |
| Female | 41 (36.3%) | 23 (26.4%) |  |  |  |  |
| Male | 72 (63.7%) | 64 (73.6%) |  |  |  |  |
| Diabetes mellitus | 26 (23.0%) | 22 (25.3%) | 0.740 | 1.133 (0.590–2.175) | — | — |
| Smoking | 25 (22.1%) | 21 (24.1%) | 0.738 | 1.120 (0.578–2.172) | — | — |
| Alcohol abuse | 6 (5.3%) | 5 (5.7%) | 1.000 | 1.087 (0.321–3.688) | — | — |
| ASA III–IV | 63 (55.8%) | 56 (64.4%) | 0.247 | 1.434 (0.807–2.547) | — | — |
| Neoadjuvant therapy | 58 (51.3%) | 53 (60.9%) | 0.198 | 1.478 (0.838–2.606) | — | — |
| Age > median | 47 (41.6%) | 51 (58.6%) | 0.022 | 1.989 (1.128–3.509) | 0.002 | 2.808 (1.468–5.374) |
| BMI > median | 68 (60.2%) | 31 (35.6%) | <0.001 | 0.366 (0.205–0.653) | 0.003 | 0.389 (0.210–0.720) |
| UICC stage III–IV | 41 (36.3%) | 56 (64.4%) | <0.001 | 3.172 (1.771–5.681) | <0.001 | 3.883 (2.030–7.427) |

**Abbreviations:** OR, odds ratio; CI, confidence interval; ASA, American Society of Anesthesiologists; BMI, body mass index; UICC, Union for International Cancer Control.

**Supplementary table 3. Patient characteristics, tumor features, treatment, and postoperative outcomes according to preoperative sarcopenia status (Reference cut-off)**

| **Variable** | **Sarcopenia negative (n=113)** | **Sarcopenia positive (SMI or PMTH or both) (n=87)** | **p** |
| --- | --- | --- | --- |
| **Gender** |  |  | 0,169 |
| Female (n, %) | 41 (36,3%) | 23 (26,4%) |  |
| Male (n, %) | 72 (63,7%) | 64 (73,6%) |  |
| **Age** (years, median, IQR) | 65,40 (57,05 – 73,75) | 73,10 (66,30 – 79,5) | < 0,001 |
| **BMI** (kg/m², median, IQR) | 26,20 (23,805 – 28,595) | 23,96 (21,65 – 26,27) | < 0,001 |
| **Risk factors** |  |  |  |
| Nicotine abuse (n, %) | 25 (22,1%) | 21 (24,1%) | 0,738 |
| Alcohol abuse (n, %) | 6 (5,3%) | 5 (5,7%) | 1,000 |
| Diabetes mellitus (n, %) | 26 (23,0%) | 22 (25,3%) | 0,740 |
| **ASA physical status** |  |  | 0,588 |
| ASA I (n, %) | 5 (4,4%) | 2 (2,3%) |  |
| ASA II (n, %) | 45 (39,8%) | 29 (33,3%) |  |
| ASA III (n, %) | 60 (53,1%) | 54 (62,1%) |  |
| ASA IV (n, %) | 3 (2,7%) | 2 (2,3%) |  |
| **Perioperative chemotherapy** |  |  |  |
| Neoadjuvant chemotherapy  (n, %) | 58 (51,3%) | 53 (60,9%) | 0,198 |
| Adjuvant chemotherapy  (n, %) | 39 (34,5%) | 23 (26,4%) | 0,280 |
| **UICC stage** |  |  | <0.001 |
| UICC stage I (n, %) | 46 (40,7%) | 17 (19,5%) |  |
| UICC stage II (n, %) | 26 (23,0%) | 14 (16,1%) |  |
| UICC stage III (n, %) | 23 (20,4%) | 34 (39,1%) |  |
| UICC stage IV (n, %) | 18 (15,9%) | 22 (25,3%) |  |
| **pT category** |  |  | 0.002 |
| pT0 (n, %) | 8 (7,1%) | 3 (3,4%) |  |
| pT1 (n, %) | 35 (31,0%) | 9 (10,3%) |  |
| pT2 (n, %) | 14 (12,4%) | 9 (10,3%) |  |
| pT3 (n, %) | 41 (36,3%) | 48 (55,2%) |  |
| pT4 (n, %) | 15 (13,3%) | 18 (20,7%) |  |
| **pN category** |  |  | 0.004 |
| pN0 (n, %) | 58 (51,3%) | 30 (34,5%) |  |
| pN1 (n, %) | 23 (20,4%) | 19 (21,8%) |  |
| pN2 (n, %) | 19 (16,8%) | 11 (12,6%) |  |
| pN3 (n, %) | 13 (11,5%) | 27 (31,0%) |  |
| **Metastatic status** |  |  | 0,198 |
| M0 (n, %) | 96 (85%) | 67 (77) |  |
| M1 (n, %) | 17 (15,0%) | 20 (23,0%) |  |
| **Resection status** |  |  | 0,077 |
| R0 (n, %) | 104 (92) | 73 (83.9) |  |
| R1/R2 resection (n, %) | 9 (8,0%) | 14 (16,1) |  |
| **Surgical procedure** |  |  | 0.632 |
| Subtotal gastrectomy (n, %) | 31 (27,4%) | 19 (21,8%) |  |
| Total gastrectomy (n, %) | 52 (46,0%) | 42 (48,3%) |  |
| Extended transhiatal gastrectomy (n, %) | 30 (26,5%) | 26 (29,9%) |  |
| **Postoperative complications** |  |  |  |
| Overall postoperative complications (n, %) | 63 (55,8%) | 48 (55,2%) | 1,000 |
| Major complications,  Clavien-Dindo grade > IIIa  (n, %) | 22 (19,5%) | 23 (26,4%) | 0,306 |
| **Type of postoperative complication** |  |  |  |
| Anastomotic leakage (n, %) | 8 (7,1%) | 12 (13,8%) | 0,154 |
| Duodenal stump leakage (n, %) | 9 (8,0%) | 1 (1,1%) | 0,045 |
| Organ/space infection (n, %) | 14 (12,4%) | 11 (12,6%) | 1,000 |
| Surgical site infection (n, %) | 14 (12,4%) | 11 (12,6%) | 1,000 |
| Pneumonia (n, %) | 8 (7,1%) | 10 (11,5%) | 0,324 |
| Respiratory insufficiency, n (%) | 25 (22,1%) | 10 (11,5%) | 0,061 |
| Other pulmonary complications  (n, %) | 28 (24,8) | 10 (11,5%) | 0,324 |
| Severe pulmonary complications  (CD > 3a) (n, %) | 20 (17,7%) | 14 (16,1%) | 0,850 |
| **Mortality** |  |  |  |
| In-hospital mortality (n, %) | 8 (7,1%) | 8 (9,2%) | 0,608 |
| 30-day mortality (n, %) | 6 (5,3%) | 5 (5,7%) | 1,000 |

**Supplementary table 4. Patient characteristics, tumor features, treatment, and postoperative outcomes according to combined preoperative sarcopenia status (Reference cut-off)**

| **Variable** | **Sarcopenia negative (n=113)** | **Sarcopenia positive (SMI and PMTH) (n=28)** | **p** |
| --- | --- | --- | --- |
| **Gender** |  |  | 0,180 |
| Female (n, %) | 41 (36,3) | 6 (21,4) |  |
| Male (n, %) | 72 (63,7) | 22 (78,6) |  |
| **Age** (years, median, IQR) | 65,4 (54.5 – 74.1) | 73,2 (65,5 – 75,9) | **0,004** |
| **BMI** (kg/m², median, IQR) | 26,2 (23,9 – 28,7) | 23.6 (19,4 – 25,4) | **<0,001** |
| **Risk factors** |  |  |  |
| Nicotine abuse (n, %) | 25 (22,1%) | 6 (21,4) | 1,000 |
| Alcohol abuse (n, %) | 6 (5,3%) | 2 (7,1) | 0,658 |
| Diabetes mellitus (n, %) | 26 (23,0%) | 5 (17,9) | 0,622 |
| **ASA status** |  |  | 0,405 |
| ASA I (n, %) | 5 (4,4%) | 1 (3,6) |  |
| ASA II (n, %) | 45 (39,8%) | 7 (25) |  |
| ASA III (n, %) | 60 (53,1%) | 19 (67,9) |  |
| ASA IV (n, %) | 3 (2,7%) | 1 (3,6) |  |
| **Perioperative chemotherapy** |  |  |  |
| Neoadjuvant chemotherapy  (n, %) | 58 (51,3%) | 16 (57,1) | 0,674 |
| Adjuvant chemotherapy  (n, %) | 39 (34,5%) | 7 (25) | 0,377 |
| **UICC stage** |  |  | 0,127 |
| UICC stage I (n, %) | 46 (40,7%) | 6 (21,4) |  |
| UICC stage II (n, %) | 26 (23,0%) | 6 (21,4) |  |
| UICC stage III (n, %) | 23 (20,4%) | 11 (39,3) |  |
| UICC stage IV (n, %) | 18 (15,9%) | 5 (17,9) |  |
| **pT category** |  |  | 0,137 |
| pT0 (n, %) | 8 (7,1%) | 1 (3,6) |  |
| pT1 (n, %) | 35 (31,0%) | 3 (10,7) |  |
| pT2 (n, %) | 14 (12,4%) | 3 (10,7) |  |
| pT3 (n, %) | 41 (36,3%) | 15 (53,6) |  |
| pT4 (n, %) | 15 (13,3%) | 6 (21,4) |  |
| **pN category** |  |  | 0,157 |
| pN0 (n, %) | 58 (51,3%) | 10 (35,7) |  |
| pN1 (n, %) | 23 (20,4%) | 8 (28,6) |  |
| pN2 (n, %) | 19 (16,8%) | 3 (10,7) |  |
| pN3 (n, %) | 13 (11,5%) | 7 (25) |  |
| **Metastatic status** |  |  | 0,772 |
| M0 (n, %) | 96 (85%) | 5 (17,9) |  |
| M1 (n, %) | 17 (15,0%) | 23 (82,1) |  |
| **Resection status** |  |  | 0,290 |
| R0 (n, %) | 104 (92) | 24 (85,7) |  |
| R1/R2 resection (n, %) | 9 (8,0%) | 4 (14,3) |  |
| **Surgical procedure** |  |  | 0,963 |
| Subtotal gastrectomy (n, %) | 31 (27,4%) | 8 (28,6) |  |
| Total gastrectomy (n, %) | 52 (46,0%) | 12 (42,9) |  |
| Extended transhiatal gastrectomy (n, %) | 30 (26,5%) | 8 (28,6) |  |
| **Postoperative complications** |  |  |  |
| Overall postoperative complications (n, %) | 63 (55,8%) | 12 (42,9) | 0,291 |
| Major complications,  Clavien-Dindo grade > IIIa  (n, %) | 22 (19,5%) | 7 (25) | 0,602 |
| **Type of postoperative complication** |  |  |  |
| Anastomotic leakage (n, %) | 8 (7,1%) | 2 (7,1) | 1,000 |
| Duodenal stump leakage (n, %) | 9 (8,0%) | 0 (0) | 0,205 |
| Organ/space infection (n, %) | 14 (12,4%) | 3 (10,7) | 1,000 |
| Surgical site infection (n, %) | 14 (12,4%) | 4 (14,3) | 0,757 |
| Pneumonia (n, %) | 8 (7,1%) | 4 (14,3) | 0,256 |
| Respiratory insufficiency, n (%) | 25 (22,1) | 2 (7,1) | 0,105 |
| Other pulmonary complications  (n, %) | 28 (24,8) | 5 (17,9) | 0,473 |
| Severe pulmonary complications  (CD > 3a) (n, %) | 20 (17,7) | 4 (14,3) | 0,785 |
| **Mortality** |  |  |  |
| In-hospital mortality (n, %) | 8 (7,1%) | 4 (14,3) | 0,256 |
| 30-day mortality (n, %) | 6 (5,3%) | 3 (10,7) | 0,382 |

**Supplementary table 5. Association between sarcopenia measures and postoperative outcomes**

| **Outcome** | **SMI (Prado)**  **n (%) / p** | **SMI (Reference)**  **n (%) / p** | **PMTH (GU)**  **n (%) / p** | **PMTH (Reference)**  **n (%) / p** |
| --- | --- | --- | --- | --- |
| Overall postoperative complications | 62 (52.1) / 0.250 | 18 (50.0) / 0.467 | 27 (56.3) / 1.000 | 42 (51.9) / 0.473 |
| Major complications, CD grade > IIIa | 24 (20.2) / 0.390 | 9 (25.0) / 0.665 | 14 (29.2) / 0.232 | 21 (25.9) / 0.306 |
| Anastomotic leakage | 12 (10.1) / 1.000 | 4 (11.1) / 0.763 | 6 (12.5) / 0.579 | 10 (12.3) / 0.346 |
| Duodenal stump leakage | 4 (3.4) / 0.322 | 0 (0.0) / 0.214 | 2 (4.2) / 1.000 | 1 (1.2) / 0.053 |
| Organ/space infection | 14 (11.8) / 0.828 | 6 (16.7) / 0.408 | 6 (12.5) / 1.000 | 8 (9.9) / 0.514 |
| Surgical site infection | 16 (13.4) / 0.669 | 6 (16.7) / 0.408 | 9 (18.8) / 0.214 | 9 (11.1) / 0.670 |
| Respiratory insufficiency | 14 (11.8) / **0.013** | 3 (8.3) / 0.146 | 8 (16.7) / 1.000 | 9 (11.1) / 0.087 |
| Pneumonia | 12 (10.1) / 0.619 | 5 (13.9) / 0.330 | 8 (16.7) / **0.041** | 9 (11.1) / 0.451 |
| Other pulmonary complications | 23 (19.3) / 0.228 | 7 (19.4) / 0.826 | 12 (25.0) / 0.691 | 15 (18.5) / 0.389 |
| Severe pulmonary complications (CD > 3a) | 16 (14.4) / 0.126 | 6 (16.7) / 1.000 | 11 (22.9) / 0.192 | 12 (14.8) / 0.572 |
| In-hospital mortality | 11 (9.2) / 0.597 | 5 (13.9) / 0.173 | 6 (12.5) / 0.218 | 7 (8.6) / 0.794 |
| 30-day mortality | 9 (7.6) / 0.205 | 3 (8.3) / 0.421 | 3 (6.3) / 0.724 | 5 (6.2) / 0.757 |

**Abbreviations:** SMI, skeletal muscle index; PMTH, psoas muscle thickness; CCI, Comprehensive Complication Index; CD, Clavien–Dindo classification.

**Supplementary table 6. Association between sarcopenia parameters and Comprehensive Complication Index (CCI)**

| **Parameter** | **n (%)** | **Mean ± SD** | **p** |
| --- | --- | --- | --- |
| SMI (Prado et al.) | 119 (59.5) | 20.65 ± 29.65 | 0.158 |
| SMI (Reference cut-off) | 36 (18.0) | 23.47 ± 32.79 | 0.835 |
| PMTH (Gu et al.) | 48 (23.7) | 24.00 ± 29.54 | 0.592 |
| PMTH (Reference cut-off) | 81 (39.9) | 21.12 ± 28.53 | 0.652 |

**Abbreviations:** SMI, skeletal muscle index; PMTH, psoas muscle thickness; CCI, Comprehensive Complication Index; SD, standard deviation

**Supplementary table 7. Association between sarcopenia parameters and length of hospital stay**

| **Parameter** | **n (%)** | **Median (IQR)** | **p** |
| --- | --- | --- | --- |
| SMI (Prado et al.) | 118 (59.3) | 14 (6.5–21.5) | 0.124 |
| SMI (Reference cut-off) | 35 (17.6) | 14 (8–20) | 0.467 |
| PMTH (Gu et al.) | 48 (23.7) | 18 (12–24) | **0.035** |
| PMTH (Reference cut-off) | 80 (39.6) | 15 (9–21) | **0.018** |

**Abbreviations:** SMI, skeletal muscle index; PMTH, psoas muscle thickness; IQR, interquartile range

**Supplementary Figure 1: Kaplan–Meier analyses of overall survival and recurrence-free survival according to combined preoperative sarcopenia status based on the reference SMI and PMTH cut-offs**

**Supplementary Figure 2. Association between preoperative sarcopenia and overall survival according to subsequent adjuvant chemotherapy**

**p interaction**

**0.775**

**0.590**

**0.961**

**0.645**

**0.987**

**Legend:** Forest plot showing hazard ratios for overall survival associated with sarcopenia among patients who received neoadjuvant chemotherapy, stratified by subsequent receipt of adjuvant chemotherapy. Hazard ratios with 95% confidence intervals are shown separately for neoadjuvant chemotherapy only and neoadjuvant plus adjuvant chemotherapy for each sarcopenia definition. The vertical dashed line indicates a hazard ratio of 1.0.

**Abbreviations:** SMI, skeletal muscle index; PMTH, psoas muscle thickness normalized to height; HR, hazard ratio; CI, confidence interval.

**Supplementary Figure 3. Kaplan–Meier analysis of overall survival according to preoperative sarcopenia among patients receiving neoadjuvant chemotherapy only**

**Legend:** Kaplan–Meier curves for overall survival according to sarcopenia defined by (a) the Prado et al. SMI cut-off, (b) the reference SMI cut-off, (c) the Gu et al. PMTH cut-off, and (d) the reference PMTH cut-off. Hazard ratios with 95% confidence intervals and p values are shown for each definition. Shaded areas indicate 95% confidence intervals. Patients at risk are shown below each panel.

**Abbreviations:** SMI, skeletal muscle index; PMTH, psoas muscle thickness normalized to height; HR, hazard ratio; CI, confidence interval.

**Supplementary Figure 3. Kaplan–Meier analysis of overall survival according to preoperative sarcopenia among patients receiving neoadjuvant and adjuvant chemotherapy**

**Legend:** Kaplan–Meier curves for overall survival according to sarcopenia defined by (a) the Prado et al. SMI cut-off, (b) the reference SMI cut-off, (c) the Gu et al. PMTH cut-off, and (d) the reference PMTH cut-off in patients who received neoadjuvant and adjuvant chemotherapy. Hazard ratios with 95% confidence intervals and p values are shown for each definition. Shaded areas indicate 95% confidence intervals. Patients at risk are shown below each panel.

**Abbreviations:** SMI, skeletal muscle index; PMTH, psoas muscle thickness normalized to height; HR, hazard ratio; CI, confidence interval.

**Supplementary Figure 5. Overall survival according to combined sarcopenia defined by the reference SMI and PMTH cut-offs, stratified by perioperative chemotherapy**

**Legend:** Kaplan–Meier curves for overall survival according to combined sarcopenia defined by both reference SMI and PMTH cut-offs among patients receiving (a) neoadjuvant chemotherapy only and (b) neoadjuvant plus adjuvant chemotherapy. Hazard ratios with 95% confidence intervals and p values are shown for each treatment group. Shaded areas indicate 95% confidence intervals. Patients at risk are shown below each panel.

**Abbreviations:** SMI, skeletal muscle index; PMTH, psoas muscle thickness normalized to height; HR, hazard ratio; CI, confidence interval.
